# Supplementary material for: Lectins and polysaccharide EPS I have flow-responsive roles in the attachment and biofilm mechanics of plant pathogenic Ralstonia
Source: PLoS Pathog. 2024 Sep 23;20(9):e1012358. doi: 10.1371/journal.ppat.1012358 (PMC11449490; doi:10.1371/journal.ppat.1012358)
Supplement: S1 Fig — A and B) Seedling root endosphere colonization. Roots of 4-day-old tomato seedlings were inoculated with 10 μL containing 104 CFU of Rps GMI1000 wild-type, ΔlecF (A, light blue) or ΔlecX (B, light green). After 48 h at room temperature, inoculated seedlings were surface sterilized and four roots were pooled per technical replicate, homogenized in sterile water, and dilution plated to quantify internal bacterial populations. Experiments were repeated three times with 9 to 12 technical replicates (Mann-Whitney test; A, P = 0.2736; B, P = 0.7110). Horizontal yellow bars indicate the geometric mean. C and D) 21-day-old tomato plants were petiole-inoculated with 2 μL containing 2000 CFU of ΔlecF (C, light blue) or ΔlecX (D, light green). Three dpi, a mid-stem sample above the point of inoculation was harvested, homogenized, and dilution plated to quantify stem colonization. Data shown represent three experiments with 11–20 plants for ΔlecF and two experiments with 12 plants each for ΔlecX (Mann-Whitney test; C, P = 0.2541; D, P = 0.3088). E-G) 21-day-old tomato plants were soil soak inoculated with 50 mL containing 5x10^6^ CFU of wild-type GMI1000, ΔlecF (E), ΔlecX (F), or ΔlecF/X (G). Disease severity was rated over 14 days on a scale from 0 (no wilting) to 4 (76–100% of plant wilting). Data reflect three experiments, each with 11–15 plants per treatment (Repeated measures Two-way ANOVA; E, P = 0.6584; F, P = 0.7637; G, P = 0.0016). (DOCX) [file ppat.1012358.s001.docx]

**Carter et al. Lectins, EPS, and Biofilms in Plant Pathogenic *Ralstonia* Supplemental Figure S1**

**
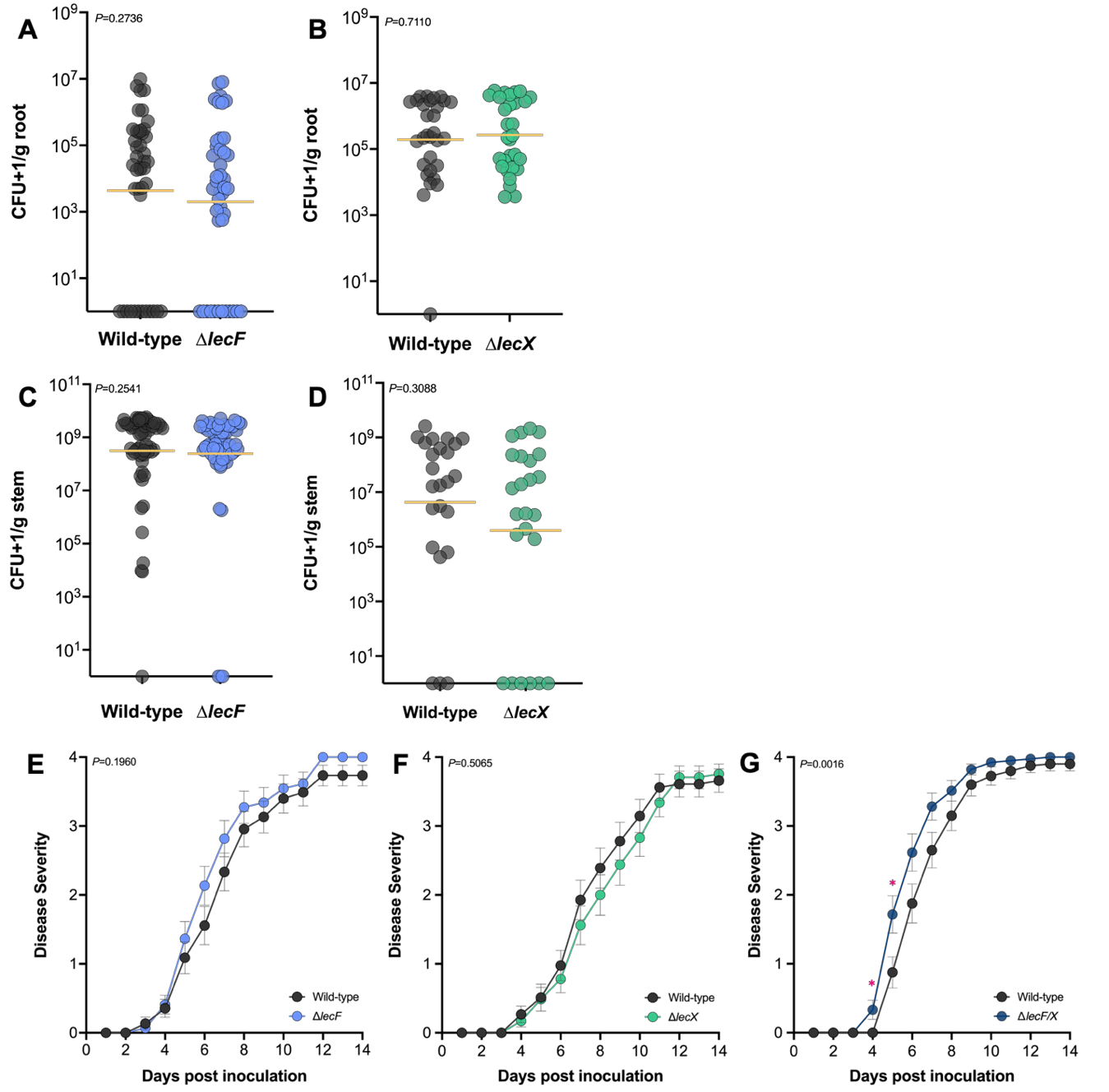
**

**Figure S1. *Rps* does not require LecF and LecX for tomato root or stem colonization or for full virulence. A** and **B) Seedling root endosphere colonization.** Roots of 4-day-old tomato seedlings were inoculated with 10 µL containing 10^4^ CFU of *Rps* GMI1000 wild-type, ∆*lecF* (A, light blue) or ∆*lecX* (B, light green). After 48 h at room temperature, inoculated seedlings were surface sterilized and four roots were pooled per technical replicate, homogenized in sterile water, and dilution plated to quantify internal bacterial populations. Experiments were repeated three times with 9 to 12 technical replicates (Mann-Whitney test; A, *P*=0.2736; B, *P*=0.7110). Horizontal yellow bars indicate the geometric mean. **C** and **D)** 21-day-old tomato plants were petiole-inoculated with 2 µL containing 2000 CFU of ∆*lecF* (C, light blue) or ∆*lecX* (D, light green). Three dpi, a mid-stem sample above the point of inoculation was harvested, homogenized, and dilution plated to quantify stem colonization. Data shown represent three experiments with 11-20 plants for ∆*lecF* and two experiments with 12 plants each for ∆*lecX* (Mann-Whitney test; C, *P*=0.2541; D, *P*=0.3088). **E-G)** 21-day-old tomato plants were soil soak inoculated with 50 mL containing 5x10^6^ CFU of wild-type GMI1000, ∆*lecF* (E), ∆*lecX* (F), or ∆*lecF/X* (G). Disease severity was rated over 14 days on a scale from 0 (no wilting) to 4 (76-100% of plant wilting). Data reflect three experiments, each with 11-15 plants per treatment (Repeated measures Two-way ANOVA; E, *P*=0.6584; F, *P*=0.7637; G, *P*=0.0016).
