# Supplementary material for: Lectins and polysaccharide EPS I have flow-responsive roles in the attachment and biofilm mechanics of plant pathogenic Ralstonia
Source: PLoS Pathog. 2024 Sep 23;20(9):e1012358. doi: 10.1371/journal.ppat.1012358 (PMC11449490; doi:10.1371/journal.ppat.1012358)
Supplement: S2 Fig — Total RNA was extracted from pellets of 10 mL log phase (OD600nm = 0.2–0.6) cultures of wild-type strain GMI1000 (WT), ΔlecF, ΔlecX, and ΔlecF/X and reverse transcribed into cDNA. Gene expression of lecF, lecM, and lecX was measured with qRT-PCR as described in Methods. Fold-changes in gene expression levels relative to those in wild-type strain GMI1000 are shown on base-2 logarithmic scale. Data shown are means of three independent replicate experiments. Asterisks indicate a difference in gene expression between the stated condition (ΔlecF, ΔlecX, or ΔlecF/X) and wild-type GMI1000 (Student’s t-test; *P≤0.05, **P≤0.01, ***P≤0.001, ****P<0.0001). (DOCX) [file ppat.1012358.s002.docx]

**Carter et al. Lectins, EPS, and Biofilms in Plant Pathogenic *Ralstonia***

**Supplemental Figure S2**

**
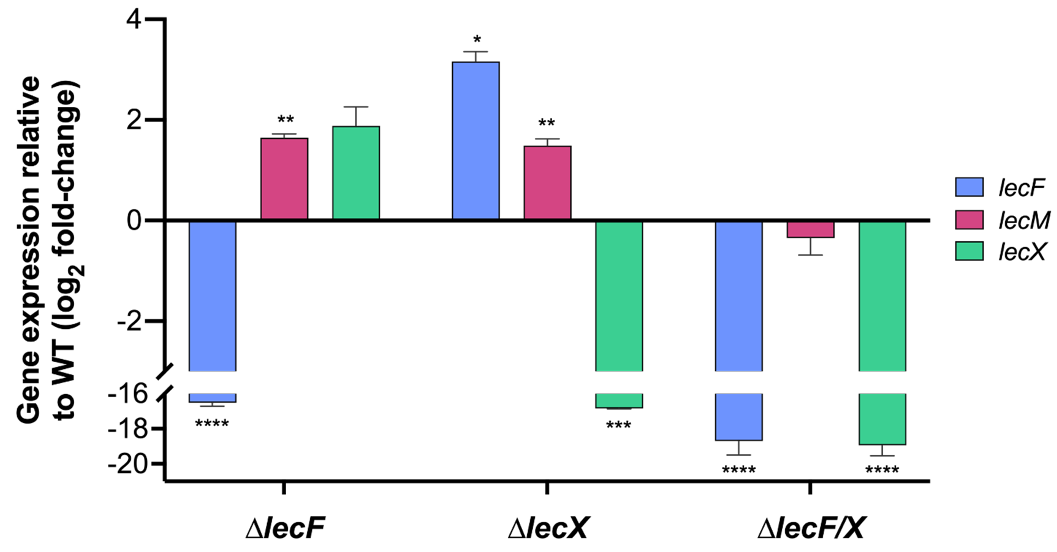
**

**Figure S2. Deletion of *Rps* *lecF* or *lecX* dysregulates the expression of the remaining two lectin genes.** Total RNA was extracted from pellets of 10 mL log phase (OD_600nm_=0.2-0.6) cultures of wild-type strain GMI1000 (WT), ∆*lecF*, ∆*lecX,* and ∆*lecF/X* and reverse transcribed into cDNA. Gene expression of *lecF*, *lecM*, and *lecX* was measured with qRT-PCR as described in Methods. Fold-changes in gene expression levels relative to those in wild-type strain GMI1000 are shown on base-2 logarithmic scale. Data shown are means of three independent replicate experiments. Asterisks indicate a difference in gene expression between the stated condition (∆*lecF*, ∆*lecX,* or ∆*lecF/X*) and wild-type GMI1000 (Student’s t-test; *P≤0.05, **P≤0.01, ***P≤0.001, ****P<0.0001).
