# Supplementary material for: Lectins and polysaccharide EPS I have flow-responsive roles in the attachment and biofilm mechanics of plant pathogenic Ralstonia
Source: PLoS Pathog. 2024 Sep 23;20(9):e1012358. doi: 10.1371/journal.ppat.1012358 (PMC11449490; doi:10.1371/journal.ppat.1012358)
Supplement: S3 Fig — Bar graph showing the percent of sequenced strains in each RSSC phylotype carrying a gene predicted to encode LecF (blue), LecM (red), and LecX (green), based on genomes of 393 RSSC strains analyzed with the Kbase protein blast function as described in Fig 3. Black numbers below the x-axis indicate the number of genomes analyzed from each phylotype. White numbers within the bars indicated the number of strains that encode the corresponding lectin. (DOCX) [file ppat.1012358.s003.docx]

**Carter et al. Lectins, EPS, and Biofilms in Plant Pathogenic *Ralstonia***

**Supplemental Figure S3**


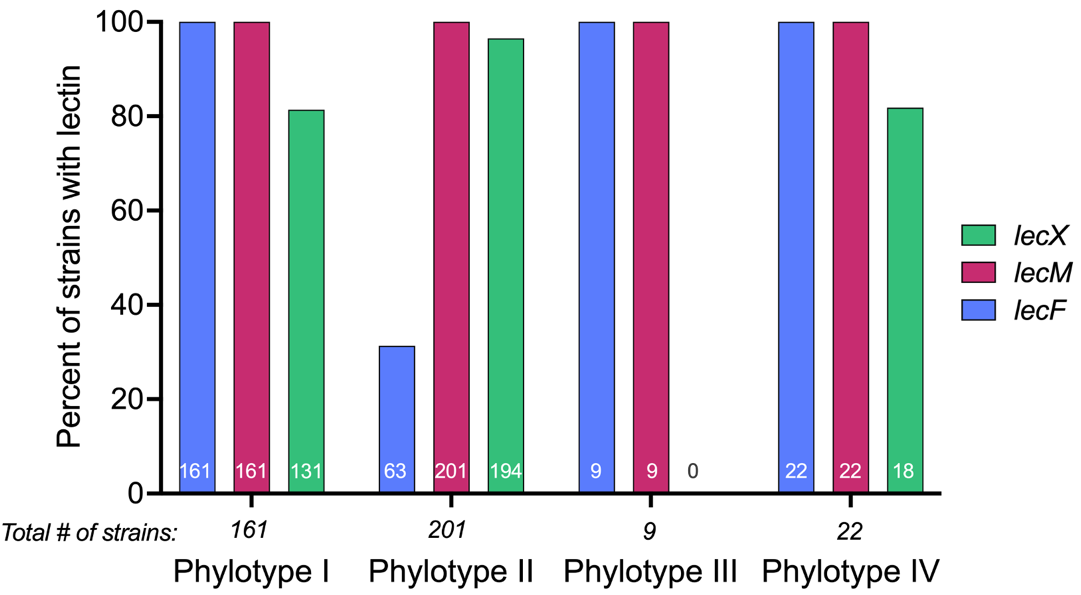


**Figure S3. Conservation of lectin genes across the RSSC.** Bar graph showing the percent of sequenced strains in each RSSC phylotype carrying a gene predicted to encode LecF (blue), LecM (red), and LecX (green), based on genomes of 393 RSSC strains analyzed with the Kbase protein blast function as described in Figure 3. Black numbers below the x-axis indicate the number of genomes analyzed from each phylotype. White numbers within the bars indicated the number of strains that encode the corresponding lectin.
