# Supplementary material for: Lectins and polysaccharide EPS I have flow-responsive roles in the attachment and biofilm mechanics of plant pathogenic Ralstonia
Source: PLoS Pathog. 2024 Sep 23;20(9):e1012358. doi: 10.1371/journal.ppat.1012358 (PMC11449490; doi:10.1371/journal.ppat.1012358)
Supplement: S4 Fig — Total RNA was extracted from log phase (OD600nm = 0.2–0.6) overnight cultures of (A) phylotype III strains CMR15, CMR15+lecX, UW386, UW386+lecX, or (B) phylotype II strains UW163, UW163+lecF, UW551, and UW551+lecF. Lectin gene expression (lecF [blue], lecM [red], lecX [green]) in the heterologous expression strains is shown relative to gene expression in their respective wild-type strains, presented as fold change on a base-2 logarithmic scale. The experiment was repeated three times. Asterisks indicate a significant difference in gene expression between the stated condition and their respective wild-type strains (Student’s t-test; *P≤0.05, **P≤0.01, ***P≤0.001). (DOCX) [file ppat.1012358.s004.docx]

**Carter et al. Lectins, EPS, and Biofilms in Plant Pathogenic *Ralstonia***

**Supplemental Figure S4**


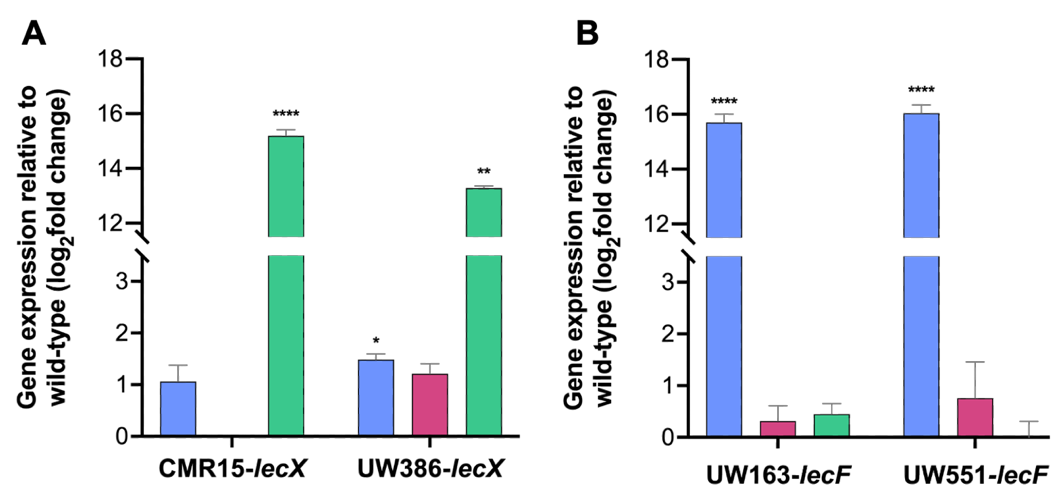


**Figure S4. Lectin gene expression in *Ralstonia* strains heterologously expressing a lectin that they naturally lack.** Total RNA was extracted from log phase (OD_600nm_=0.2-0.6) overnight cultures of (A) phylotype III strains CMR15, CMR15+*lecX*, UW386, UW386*+lecX,* or (B) phylotype II strains UW163, UW163+*lecF*, UW551, and UW551*+lecF.* Lectin gene expression (*lecF* [blue], *lecM* [red], *lecX* [green]) in the heterologous expression strains is shown relative to gene expression in their respective wild-type strains, presented as fold change on a base-2 logarithmic scale. The experiment was repeated three times. Asterisks indicate a significant difference in gene expression between the stated condition and their respective wild-type strains (Student’s t-test; *P≤0.05, **P≤0.01, ***P≤0.001).
