# Supplementary material for: Lectins and polysaccharide EPS I have flow-responsive roles in the attachment and biofilm mechanics of plant pathogenic Ralstonia
Source: PLoS Pathog. 2024 Sep 23;20(9):e1012358. doi: 10.1371/journal.ppat.1012358 (PMC11449490; doi:10.1371/journal.ppat.1012358)
Supplement: S5 Fig — A and B) 150 μL of 107 CFU/mL wild-type Ralstonia GMI1000, ΔlecF, ΔlecX, and ΔlecF/X resuspended in CPG were aliquoted into polystyrene microtiter plates and incubated in a BioTek plate reader at 28°C. and growth was measured as OD600. The data shown reflect 2–4 experiments, with 6 technical replicates each. The area under the growth curve was calculated and plotted in B (ANOVA, P = 0.4933). C and D) 150 μL of 107 CFU/mL UW163, UW163+lecF, UW386, and UW386+lecX resuspended in CPG were aliquoted into polystyrene microtiter plates. Plates were grown in a BioTek plate reader at 28°C and growth was measured as OD600. Data shown reflect 3 experiments, with 6–12 technical replicates each. The area under the growth curve was calculated and plotted in D (T-test, P = 0.6029, P = 0.0129). (DOCX) [file ppat.1012358.s005.docx]

**Carter et al. Lectins, EPS, and Biofilms in Plant Pathogenic *Ralstonia***

**Supplemental Figure S5**

**
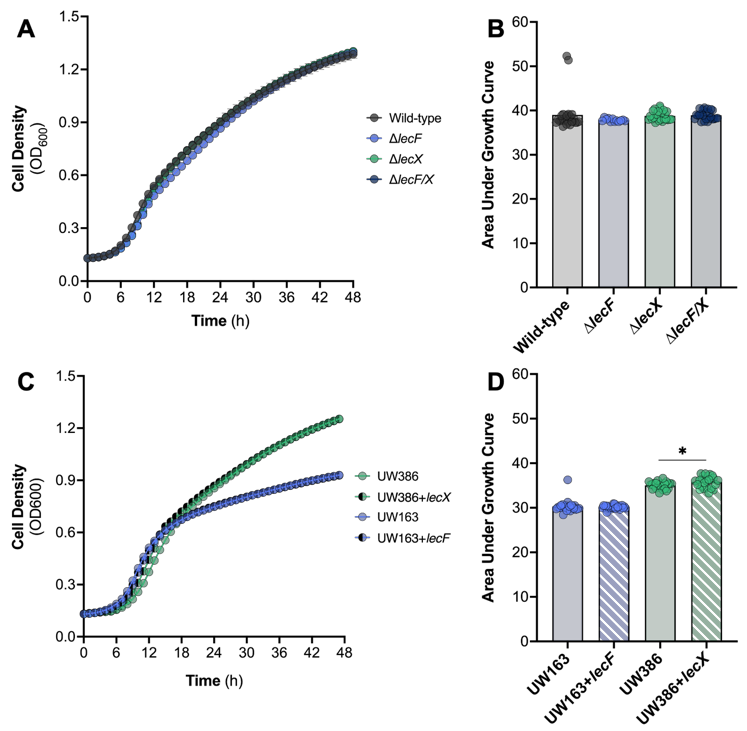
**

**Figure S5. Growth dynamics of lectin mutants in CPG rich media. A** and **B**) 150 μL of 10^7^ CFU/mL wild-type *Ralstonia* GMI1000, ∆*lecF*, ∆*lecX*, and ∆*lecF/X* resuspended in CPG were aliquoted into polystyrene microtiter plates and incubated in a BioTek plate reader at 28°C. and growth was measured as OD_600_. The data shown reflect 2-4 experiments, with 6 technical replicates each. The area under the growth curve was calculated and plotted in **B** (ANOVA, *P*=0.4933). **C** and **D**) 150 μL of 10^7^ CFU/mL UW163, UW163+*lecF*, UW386, and UW386+*lecX* resuspended in CPG were aliquoted into polystyrene microtiter plates. Plates were grown in a BioTek plate reader at 28°C and growth was measured as OD_600_. Data shown reflect 3 experiments, with 6-12 technical replicates each. The area under the growth curve was calculated and plotted in **D** (T-test, *P*=0.6029, *P*=0.0129).
