# Supplementary material for: Lectins and polysaccharide EPS I have flow-responsive roles in the attachment and biofilm mechanics of plant pathogenic Ralstonia
Source: PLoS Pathog. 2024 Sep 23;20(9):e1012358. doi: 10.1371/journal.ppat.1012358 (PMC11449490; doi:10.1371/journal.ppat.1012358)
Supplement: S1 Table — (DOCX) [file ppat.1012358.s006.docx]

**Carter et al. Lectins, EPS, and Biofilms in Plant Pathogenic *Ralstonia***

**Supplemental Table 1. Bacterial strains, plasmids, and primers**

| NAME | CHARACTERISTICS | SOURCE |
| --- | --- | --- |
| **Strains** |  |  |
| *E. coli* TOP10 | F- mcrA Δ(mrr-hsdRMS-mcrBC) Φ80lacZΔM15 ΔlacX74 recA1 araD139 Δ(ara-leu)7697 galU galK rpsL (Str^r^) endA1 nupG | Life Technologies |
| *R. pseudosolanacearum* GMI1000 (*Rps*) | Wild-type tomato isolate from French Guyana, phylotype I sequevar 18 | [1] |
| ∆*lecF* | GMI1000 carrying unmarked deletion of *rsc2107* | This study |
| ∆*lecX* | GMI1000 carrying unmarked deletion of *rsp0569* | This study |
| *∆lecF/X* | GMI1000 carrying unmarked deletions of *rsp0569* and *rsc2107* | This study |
| ∆*epsB* | An unmarked deletion of GMI1000 *rsp1018* | This study |
| *R. solanacearum* UW551 | Wild-type geranium isolate from Kenya, phyl IIB, seq1; naturally lacks *lecF* | [2] |
| UW551-*lecF* | UW551 with pUC18-miniTn7T-*lecF_GMI1000_* integrated into chromosome *att* site, Gm^R^ | This study |
| *R. solanacearum* UW163 | Wild-type plantain isolate from Peru, phyl IIB, seq 4; naturally lacks *lecF;* also named S256 | Allen lab collection |
| UW163-*lecF* | UW163 with pUC18-miniTn7T- *lecF_GMI1000_* integrated into chromosome *att* site, Gm^R^ | This study |
| *R. pseudosolanacearum* UW386 | Wild-type soil isolate from Nigeria, phyl III seq 23; naturally lacks *lecX* | [3] |
| UW386-*lecX* | UW386 with pUC18-miniTn7T-*lecX_GMI1000_* integrated into chromosome *att* site, Gm^R^ | This study |
| *R. pseudosolanacearum* CMR15 | Wild-type tomato isolate from Cameroon, phyl III seq 29; naturally lacks *lecX* | [4] |
| CMR15-*lecX* | CMR15 with pUC18-miniTn7T-*lecX* integrated into chromosome *att* site, Gm^R^ | This study |
| ∆*lecF+lecF* | ∆*lecF* with pRCK-*lecF* integrated into chromosome *att* site, Kan^R^ | This study |
| ∆*lecX+lecX* | ∆*lecX* with pRCK-*lecX* integrated into chromosome *att* site, Kan^R^ | This study |
| **Plasmids in *E. coli*** |  |  |
| pUFR80 | Positive selection (*sacB*) suicide vector; Suc^S^, Kan^R^ | [5] |
| pUFR80∆*lecF* | Vector used to generate an unmarked *lecF* mutant; Suc^S^, Kan^R^ | This study |
| pUFR80∆*lecX* | Vector used to generate an unmarked *lecX* mutant; Suc^S^, Kan^R^ | This study |
| pUFR80∆*epsB* | Vector used to generate an unmarked *epsB* mutant; Suc^S^, Kan^R^ | This study |
| pRCK-GWY | Gateway vector that integrates into the GMI1000 chromosome downstream of *glmS* in the neutral *att* site, Kan^R^ | [6] |
| pRCK-*lecF* | Vector used to integrate *lecF* into chromosome *att*site, Kan^R^ | This study |
| pRCK-*lecX* | Vector used to integrate *lecX* into chromosome *att*site, Kan^R^ | This study |
| pTNS1 | Helper vector for pUC18-miniTn7t-Gm, encodes the site-specific TnsABCD transposase, Amp^R^ | [7] |
| pUC18-miniTn7T-Gm | Vector that integrates into selectively neutral *att* site in bacterial chromosomes, Gm^R^ | [7] |
| pUC18-miniTn7T-*lecF* | pUC18-miniTn7t-Gm with *lecF*_GMI1000_ and the native promoter, Gm^R^ | This study |
| pUC18-miniTn7T-*lecX* | pUC18-miniTn7t-Gm with *lecX*_GMI1000_ and the native promoter, Gm^R^ | This study |
| **Primers** |  |  |
| pUFR80∆*lecF_*up-F | taaaacgacggccagtgccaGATCTCTGCGCCGTACAC | This study |
| pUFR80∆*lecF_*up-R | tccacattgcCTTACTGCACCGCACCAATG | This study |
| pUFR80∆*lecF_*down-F | gtgcagtaagGCAATGTGGAATCCGTTGG | This study |
| pUFR80∆*lecF_*down-R | agtcgacctgcaggcatgcaCAATACACCGTTGGCGGATG | This study |
| pUFR80∆*lecX_*up-F | tggttcgtggCGCAAAAATCAGGGCGCATC | This study |
| pUFR80∆*lecX_*up-R | attcgagctcggtacccgggTCTCGGCGTCGTTGAATC | This study |
| pUFR80∆*lecX_*down-F | taaaacgacggccagtgccaGAGATACGGGGGCAAATC | This study |
| pUFR80∆*lecX_*down-R | gatttttgcgCCACGAACCATGCAAACC | This study |
| pUFR80∆*epsB_*up-F | taaaacgacggccagtgccaGGACAGCAAGGTCTTCGTG | This study |
| pUFR80∆*epsB_*up-R | gctgcagccgCAGGGTCGTATTCCGTGC | This study |
| pUFR80∆*epsB_*down-F | tacgaccctgCGGCTGCAGCGCGGCGAA | This study |
| pUFR80∆*epsB_*down-R | agtcgacctgcaggcatgcaGCACATTGGGATTCAGGTGCACGG | This study |
| pRCK-*lecF*-F | cctagggttaacggtacACCGATGCGACCTCTTGTAG | This study |
| pRCK-*lecF*-R | gaccctagtctaagatcttTCAGTTGGTGGAGGTGTAG | This study |
| pRCK-*lecX*-F | cctagggttaacggtacCATCAGGCGAAGCATGTGAC | This study |
| pRCK-*lecX*-R | gaccctagtctaagatcttTCAGTTCCAGCCGTAGACC | This study |
| qPCR-*lecF*-F | GTTCAGACCGCTGCCACTTC | This study |
| qPCR-*lecF*-R | CGTTGAAGGCACCCGTGTA | This study |
| qPCR-*lecM*-F | CCTGGATGGTCTGGGTGTTC | This study |
| qPCR-*lecM*-R | AGGTGTATTCACGCTTCCCG | This study |
| qPCR-*lecX*-F | TTGGCATAGCCGGGTTCATT | This study |
| qPCR-*lecX*-R | TACGGATACTCGGAGGCCAA | This study |
| qPCR-*rplM*-F | CCGCAAAGCCCCATGA | [8] |
| qPCR-*rplM*-R | TGTCCGTCGCGTCAATCA | [8] |
| qPCR-*serC*-F | TGTCCGTCGCGTCAATCA | [9] |
| qPCR-*serC*-R | TCAACGCCGACGATGGT | [9] |
| qPCR-*epsB*-F | GACCGATGCCAACGCCGAG | [9] |
| qPCR-*epsB*-R | CGCGCCGATCAGCGGAAAGT | [9] |
| qPCR-*iolG*-F | GCCTGAATGCCGAGGTGTA | [10] |
| qPCR- *iolG-*R | AGGACAAATTCCTCGTGCGT | [10] |

**Supplemental Table 1 References**

1. Boucher C, Martinel A, Barberis P, AHoing G, Zisehek C. Virulence genes are carried by a megaplasmid of the plant pathogen *Pseudomonas solanacearum*. Molecular Genetics and Genomics. 1986;205: 270–275.

2. Williamson L, Nakaho K, Hudelson B, Allen C. *Ralstonia solanacearum* Race 3, Biovar 2 Strains Isolated from Geranium Are Pathogenic on Potato. Plant Dis. 2002;86: 987–991.

3. Steidl OR, Truchon AN, Hayes MM, Allen C. Complete genome resources for *Ralstonia* bacterial wilt strains UW763 (Phylotype I); Rs5 and UW700 (Phylotype II); and UW386, RUN2474, and RUN2279 (Phylotype III). Molecular Plant-Microbe Interactions. 2021;34: 1212–1215. doi:10.1094/MPMI-04-21-0086-A

4. Remenant B, Coupat-Goutaland B, Guidot A, Cellier G, Wicker E, Allen C, et al. Genomes of three tomato pathogens within the *Ralstonia solanacearum* species complex reveal significant evolutionary divergence. BMC Genomics. 2010;11: 379.

5. Castañeda A, Reddy JD, El-Yacoubi B, Gabriel DW. Mutagenesis of all eight avr genes in *Xanthomonas campestris* pv. *campestris* had no detected effect on pathogenicity, but one avr gene affected race specificity. Molecular Plant Microbe Interactions. 2005;18: 1306–1317. doi:10.1094/MPMI

6. Monteiro F, Solé M, van Dijk I, Valls M. A chromosomal insertion toolbox for promoter probing, mutant complementation, and pathogenicity studies in *Ralstonia solanacearum*. Molecular Plant Microbe Interactions. 2012;25: 557–568. doi:10.1094/MPMI-07-11-0201/SUPPL_FILE/MPMI-07-11-0201E4.PDF

7. Choi KH, Gaynor JB, White KG, Lopez C, Bosio CM, Karkhoff-Schweizer RAR, et al. A Tn7-based broad-range bacterial cloning and expression system. Nat Methods. 2005;2: 443–448. doi:10.1038/nmeth765

8. Monteiro F, Genin S, van Dijk I, Valls M. A luminescent reporter evidences active expression of *Ralstonia solanacearum* type III secretion system genes throughout plant infection. Microbiology (United Kingdom). 2012;158: 2107–2116. doi:10.1099/mic.0.058610-0

9. Khokhani D, Lowe-Power TM, Tran TM, Allen C. A single regulator mediates strategic switching between attachment/spread and growth/virulence in the plant pathogen *Ralstonia solanacearum*. mBio. 2017;8: e00895-17. doi:10.1128/mBio.00895-17

10. Carter MD, Khokhani D, Allen C. Cell density-regulated adhesins contribute to early disease development and adhesion in *Ralstonia solanacearum*. Appl Environ Microbiol. 2023;89. doi:10.1128/aem.01565-22
